# Lifelong Adaptation of Gastric Cell Proliferation and Mucosa Structure to Early Weaning-Induced Effects

**DOI:** 10.3389/fphys.2021.721242

**Published:** 2021-09-13

**Authors:** Kethleen Mesquita da Silva, Isadora Campos Rattes, Gizela Maria Agostini Pereira, Patrícia Gama

**Affiliations:** Department of Cell and Developmental Biology, Institute of Biomedical Sciences, University of São Paulo, São Paulo, Brazil

**Keywords:** cell proliferating zone, gastric mucosa, breastfeeding, early weaning, p27

## Abstract

The gastric mucosa is disturbed when breastfeeding is interrupted, and such early weaning (EW) condition permanently affects the differentiation of zymogenic cells. The aim of the study was to evaluate the immediate and long-term effects of EW on gastric cell proliferation, considering the molecular markers for cell cycle, inflammation, and metaplasia. Overall, we investigated the lifelong adaptation of gastric growth. Wistar rats were divided into suckling-control (S) and EW groups, and gastric samples were collected at 18, 30, and 60 days for morphology, RNA, and protein isolation. Inflammation and metaplasia were not identified, but we observed that EW promptly increased Ki-67-proliferative index (PI) and mucosa thickness (18 days). From 18 to 30 days, PI increased in S rats, whereas it was stable in EW animals, and such developmental change in S made its PI higher than in EW. At 60 days, the PI decreased in S, making the indices similar between groups. Spatially, during development, proliferative cells spread along the gland, whereas, in adults, they concentrate at the isthmus-neck area. EW pushed dividing cells to this compartment (18 days), increased PI at the gland base (60 days), but it did not interfere in expression of cell cycle molecules. At 18 days, EW reduced *Tgfβ2*, *Tgfβ3*, and *Tgfbr2* and TβRII and p27 levels, which might regulate the proliferative increase at this age. We demonstrated that gastric cell proliferation is immediately upregulated by EW, corroborating previous results, but for the first time, we showed that such increased PI is stable during growth and aging. We suggest that suckling and early weaning might use TGFβs and p27 to trigger different proliferative profiles during life course.

## Introduction

The gastric corpus mucosa is lined with epithelial cells that cover the surface and enter the lamina propria to form tubular glands, which contain five populations that secrete neutral and acid glycoproteins, produce HCl, synthesize pepsinogen, intrinsic factor and hormones (Karam and Leblond, 1993b; Li et al., 1996; Sáenz and Mills, 2018). Unlike the small intestine and the gastric antrum region, which have well-defined stem cell markers, the corpus area still lacks a clear characterization of these cells and the components of their niche. Previous studies using electron microscopy, pulse chasing with radioactive thymidine (Karam and Leblond, 1993a,b) and transgene mutation (Bjerknes and Cheng, 2002) have shown that proliferative cells are found at the isthmus-neck interface in the gastric gland in adult mice, and the cells derived from progenitors migrate bidirectionally toward the foveola and the base of the gland. Others have attempted to identify the cell markers for these progenitor cells (Arnold et al., 2011; Hayakawa et al., 2015; Choi et al., 2017; Matsuo et al., 2017; Yoshioka et al., 2019), but none of them detected exclusive molecules in the isthmus region (Burclaff and Mills, 2018; Han et al., 2019). More recently, it has been reported that the corpus gland is compartmentalized in two niches of independent stem cells: one at the isthmus with rapidly cycling cells and another one at the base, with slow-cycling cells (Han et al., 2019; Burclaff et al., 2020). Meanwhile, most studies focus on the description of adult stem cells and their homeostasis, and little is known about the distribution and organization of stem cell niches during the postnatal development.

Throughout development, milk-born peptides, together with growth factors synthesized in the gastric gland, contribute to regulate cell cycle and proliferation (de Andrade Sá et al., 2003; Osaki et al., 2010, 2011; Osaki and Gama, 2013; Fiore et al., 2014). Previously, we demonstrated that TGFβ is one of these key regulators for stomach growth (de Andrade Sá et al., 2003, 2008). During the suckling period, TGFβ activity is balanced and controls p27 levels (de Andrade Sá et al., 2003; Fiore et al., 2014), but if feeding pattern changes, signaling is disrupted and p27 is degraded more rapidly, increasing the epithelial renewal (de Andrade Sá et al., 2003; Fiore et al., 2014).

The interruption of breastfeeding through early weaning immediately increases gastric cell proliferation (Gama and Alvares, 2000; Ghizoni et al., 2014) as a response to EGFR cascade (Osaki et al., 2011; Osaki and Gama, 2013), but the interaction with corticosterone (Ghizoni et al., 2014) and ghrelin pathways (Bittar et al., 2016) is also discussed. Additionally, when the proliferative stimulus is evaluated as a trigger for cell differentiation, mucous neck cells, and zymogenic populations are increased in developing young-adult rats, and, for these developmental stages, such regulation is dependent on corticosterone activity (Osaki et al., 2010; Zulian et al., 2017). Importantly, in the adult gastric mucosa, the distribution of zymogenic cells remains high in early weaned rats (Teles Silva et al., 2020). When the recent studies are combined, they do not show irregular growth, erosion, and restitution failure after EW, as it had been demonstrated before (Ackerman et al., 1975, 1978; Glavin and Pare, 1985), but they do not evaluate whether there would be long-lasting effects to predispose the stomach for inflammation or lesions.

Breastfeeding maintenance is essential to growth (Lin et al., 1998; Tang et al., 1999; Gama et al., 2000; Marion et al., 2005; de Andrade Sá et al., 2008; Osaki et al., 2010; Rogier et al., 2014; Bittar et al., 2016; Zulian et al., 2017), and its premature interruption can affect gastric development; we had two main goals in this study: (1) to evaluate whether the proliferative stimulus of early weaning would be lifelong detected and how it would be associated with the distribution of dividing cells in the gland, and (2) to identify the molecules of TGFβ cascade and cell cycle response and their behavior in the gastric mucosa after breastfeeding interruption.

## Materials and Methods

### Animals and Early Weaning

Specific-pathogen-free Wistar rats were obtained from the Animal Facility at the Institute of Biomedical Sciences (University of São Paulo). Pregnant females were kept in individual cages at 22°C under 12:12-h light/dark cycle with free access to food (Nuvilab CR-1, Quimtia) and water. Delivery was set as day 0, and litters were adjusted to 9 pups per dam on the second postnatal day.

Early weaning protocol was followed as previously described (Osaki et al., 2010). At 15 days, male and female pups were randomly assigned into the suckling-control group (S) that remained with the dam until regular weaning (21 days) and the early weaned group (EW) that was transferred into another box, where hydrated powdered chow and water were offered *ad libitum*. Two times a day, EW rats received food and water through disposable Pasteur pipettes and were massaged to facilitate excretion, mimicking maternal care. The number of pups with the dam was controlled to avoid obesity, and the experiments proceeded when the body mass was observed to change according to the patterns previously established (Teles Silva et al., 2020).

Gastric samples were collected at 18 (postnatal growth), 30 (differentiated mucosa in young adults), and 60 days (adult mucosa) to allow the comparison of cell proliferation and morphology in different periods of growth and function. The screening of regulatory molecules was performed at 18 and 60 days, which were defined as two contrasting moments in cell proliferation control (Supplementary Figure 1). For sampling, the animals were euthanized after anesthesia with ketamine and xylazine chlorhydrates at 1:1 (v/v) (0.5 ml/100 g body weight) at 10:00. The stomachs were collected, opened along the lesser curvature. For morphology, after rinsing in 0.9% saline, the wall was stretched on a cork with mucosa side up for fixation in 10% formaldehyde. For RNA and protein isolation, the mucosa was scraped and added, respectively, to 100 μl of RNALater^®^ (Life Technologies) or 10 mM phenylmethylsulfonyl fluoride (PMSF) in 20-mM Tris-buffered saline (TBS).

### Morphology and Cell Proliferation

After 12 h of fixation, gastric strips were embedded in paraffin and non-serial 6-μm sections were either stained with HE for morphological evaluation or used for immunohistochemistry.

The morphology was studied under light microscopy (X20, Olympus microscope BX51), and measurements from gland depth and mucosa thickness were analyzed with Image Pro-Plus software (version 5.1.2, Media Cybernetics). Ten to fifteen fields/animal were considered.

To immunolabel Ki-67, after rehydration and peroxidase blocking, sections were transferred to 10-mM citric acid (pH 6.0) and microwaved (2 × 5 min at 600 W and 2 × 3 min at 240 W). Non-specific binding was blocked with 20% normal goat serum, and sections were incubated with monoclonal rabbit anti-Ki-67 (Supplementary Table 1). The reaction was developed by streptavidin-biotin complex (5 μg/ml, Jackson ImmunoResearch Labs.), followed by 0.07% 3.3′-diaminobenzidine (DAB) in 20-mM TBS, containing 3% hydrogen peroxide. Tissue sections were counterstained with 0.1% Mayer's hematoxylin and observed under light microscopy. Negative controls were obtained by the omission of the primary antibody.

Cell proliferation was assessed in longitudinal sections of the corpus region of the stomach, and it was estimated along the gland depth for 18-day-old rats and in the isthmus area, which is the active compartment, for 30- and 60-day-old animals, as described before (Gama and Alvares, 2000; Ghizoni et al., 2014). To determine the PI/animal, Ki-67 immunolabeled epithelial cells were counted in a total of 2,500 cells (Aherne et al., 1977; Osaki et al., 2011) under light microscopy (X100, Nikon, Japan), using an integrative eyepiece (X8, Kpl2, Zeiss, Germany).

As we were also interested in the distribution of PI according to mucosa compartments and the active and inactive niches, we evaluated them according to three segments in the gland as upper, middle, and bottom (18 days), and isthmus, neck, and base (30 and 60 days). The distinction among ages was based on differences of maturation of the gland (Ghizoni et al., 2014). To that, we determined the LI/segment, considering a total of 2,500 cells/animal, as described above.

### Gene Expression

Quantitative reverse transcription polymerase chain reaction (RT-qPCR) was used, and methods were followed as described before (Teles Silva et al., 2020) for scraped samples from gastric mucosa. Briefly, after RNA isolation (TRIzol combined with PureLink^®^ RNA Mini Kit, Invitrogen) and quantification (NanoDrop, Thermo Fisher Scientific), 3 μg of RNA were used for cDNA synthesis with the Superscript III Reverse Transcriptase enzyme (200 U/μl, Invitrogen). The genes were studied using Taqman^®^ probe assays (Thermo Fisher Scientific) (Supplementary Table 2), and 40 ng of cDNA were amplified in a Step One Plus thermocycler (Applied Biosystems). The Taqman^®^ assay for *Actb* was taken as reference. The fluorescence emitted by annealing the samples to the primers was measured at a wavelength of 470 nm, and relative quantities were calculated using the 2^−ΔΔCt^ method (Schmittgen and Livak, 2008).

### Protein Levels

Protein was extracted with a RIPA buffer (50-mM Tris-HCl, 150-mM NaCl, 1% NP-40, 1% sodium deoxycholate acid), containing protease and phosphatase inhibitors, and lysates were quantified according to Bradford's method(Bradford, 1976). Proteins were fractioned with 12% SDS-polyacrylamide gels (2 h, 100 V, 50 mA), and samples were transferred to nitrocellulose membranes (Hybond ECL, GE Healthcare), which were then stained in 0.5% Ponceau solution for quality control.

For immunoblotting, the membranes were blocked, washed, and incubated with primary antibodies (Supplementary Table 1), which were developed with secondary antibodies conjugated to peroxidase (Jackson ImmunoResearch Labs) and ECL kit (GE Healthcare). β-actin was used as internal loading control. Bands were recorded on X-ray films or by image capture with GBox transillumination system (Syngene), and densitometry was analyzed with Image J (public domain software, 1.37v, National Institute of Mental Health, NIH).

### Distribution of Smads in the Gastric Mucosa

The protocol used for the immunostaining of Smad 2/3 and Smad 2P was the same detailed above, and primary rabbit monoclonal antibodies against them were used (Supplementary Table 1). The labeling indices (LI) were determined in the gastric mucosa after counting immunostained cells in a total of 1,000 epithelial cells (X100; integrative eyepiece, X8, Kpl2, Zeiss, Germany). The distribution of Smads in the segments of the gland was observed as the LI in the upper and lower areas (18 days) and the isthmus neck and base (60 days), and it was obtained by counting the number of immunolabeled cells/segment in a total of 1,000 positive cells/animal.

Because Smad 2P translocates from the cytoplasm to the nucleus (Schmierer and Hill, 2005), we determined the intracellular location: cytoplasm, cytoplasm + nucleus, and nucleus (de Andrade Sá et al., 2008) after counting 500epithelial cells immunostained for Smad 2P.

### Statistical Analyses

Results were represented individually through the dispersion for each parameter and by bars with means ± SD. Differences between groups in each age were compared by unpaired Student's *t*-test, and differences were considered significant when *p* < 0.05. Two-way ANOVA was used to evaluate the interaction between parameters and whether the effect of EW is dependent on age (Graph Pad Prism version 9.0 for Windows). Comparative analyses between proliferative index (PI) and morphological analyses were performed with RStudio software 1.0.153 (RStudio: Integrated Development Environment for R, Boston, MA, USA).

## Results

### Early Weaning: Proliferative Compartments, Gastric Mucosa Growth, and Structure

**Figure 1:**
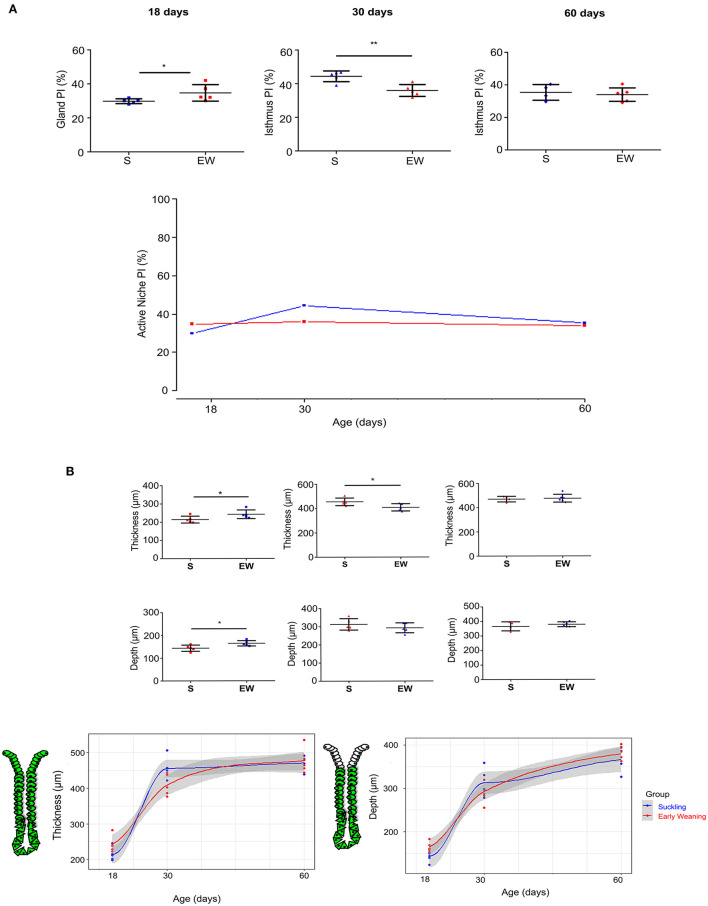
Early weaning induces changes in cell proliferation in the gastric mucosa. **(A)** Proliferative index (PI) (%) after Ki- 67, counting at 18, 30, and 60 days. Results are represented individually: S (suckling, blue) and EW (early weaning, red) and by means ± SD. ^*^p < 0.05 and ^**^p < 0.01 EW vs. the S group at the same age after Student's *t*-test. **(B)** Left: The thickness of mucosa and gland depth were measured at 18, 30, and 60 days. The thickness was evaluated by measuring the distance from the base of the gland to the epithelial surface, and depth was obtained by measuring the distance from the base of the gland to the isthmus region. Results are represented individually for S (blue) and EW (red) and by means ± SD. After Student's *t-*test: ^*^p < 0.05, *n* = 10–15 fields/animal. Right: Growth curves were used to compare the growth of mucosa and gland depth between the S and EW groups at each age.

By using HE-stained sections, we found that the cellular and histological aspects in the mucosa and submucosa layers were well-preserved in samples from S and EW groups (Supplementary Figure 2A). After Ki-67 immunolabeling, we recorded proliferative cells dispersed along the gland at 18 days (Osaki et al., 2010; Ghizoni et al., 2014). We determined the PI inside this compartment, and we found that EW increased cell proliferation in 16.4% when compared with the S group (*p* < 0.01) (Figure 1A). At 30 and 60 days, the PI was estimated in the active isthmus niche (Han et al., 2019). From 18 to 30 days, we observed that the PI increased in S rats (*p* < 0.05) (Figure 1A), whereas, in EW animals, it was maintained at the index detected at 18 days. This developmental change in the S group rendered a comparative reduction of EW PI (19%) in 30-day-old rats (*p* < 0.01). At 60 days, the PI in S rats decreased to an index similar to the EW group (Figure 1A). The overall analyses between S and EW indicated that the immediate increase of PI in response to EW flattened the curve throughout aging, as the cell proliferation was high from the beginning, at 18 days (Figure 1A). Therefore, these results suggest that the gastric mucosa kept constant PI after EW as indices were not altered by aging.

When we studied the morphometric parameters, we observed that the mucosa thickness and gland depth increased after EW in 18-day-old rats (*p* < 0.05) (Figure 1B), whereas, at 30 days, as the thickness in S increased, when groups were compared, the parameter seemed reduced by EW (*p* < 0.05) without changes in gland depth (Figure 1B). At 60 days, we did not record effects (Figures 1A,B).

**Figure 2:**
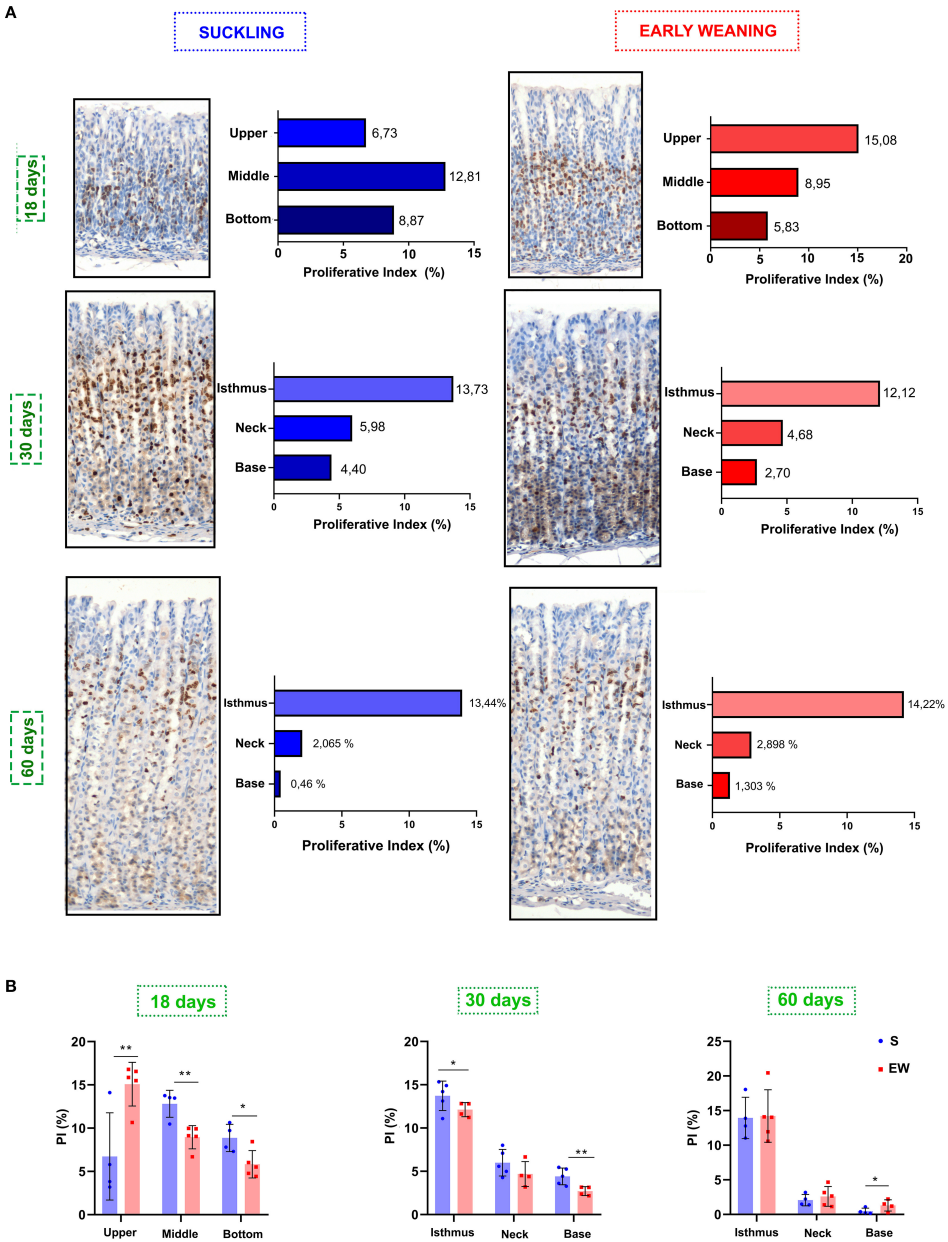
Ki-67 distribution in the gastric mucosa. **(A)** The gland areas were delineated at 18 (as upper, middle, and bottom), 30 and 60 days (as isthmus, neck, and base). Bar graphs show the distribution of Ki-67, and results are represented by means for S (blue bar) and EW (red bar). **(B)** Comparison of the distribution of proliferative cells among S and EW. Each point represents the result obtained in an animal, and the lines indicate the mean ± SD. ^*^p < 0.05; ^**^p < 0.01 (*n* = 5–6/group).

Because we previously demonstrated that, in pups, EW immediately pushes the active proliferative niche to the isthmus-neck area (Ghizoni et al., 2014), accelerating the pattern of mature glands, we investigated the distribution of proliferative cells in the different mucosa compartments. At 18 days, we found that the highest concentrations of proliferative cells were in the middle (12.81%) and bottom regions (8.87%) of the gland in the S group (Figure 2A), whereas, in the EW group, the distribution was dislocated to the upper (15.08%) and middle (8.95%) regions (Figure 2A). Accordingly, we registered changes in the segments with a significant increase (upper) and decrease (middle and bottom) of PI (*p* < 0.05) after EW (Figures 2A,B). At 30 days, we observed that the PI was concentrated in the isthmus (S = 13.73%; EW = 12.12%) (Figures 2A,B), and a significant reduction was detected at the base after EW (*p* < 0.05) (Figure 2B). At 60 days, although the same trend was registered (Figure 2A), the PI at the base of glands doubled in EW (1.1%) when compared with S (0.46%) (*p* < 0.05) (Figures 2A,B). So, we demonstrated that, during development, the change of the breastfeeding program through EW pushed proliferative cells into the main compartment, although cells remained cycling at the base of adult glands, which might make the secondary niche susceptible to environmental factors.

**Figure 3:**
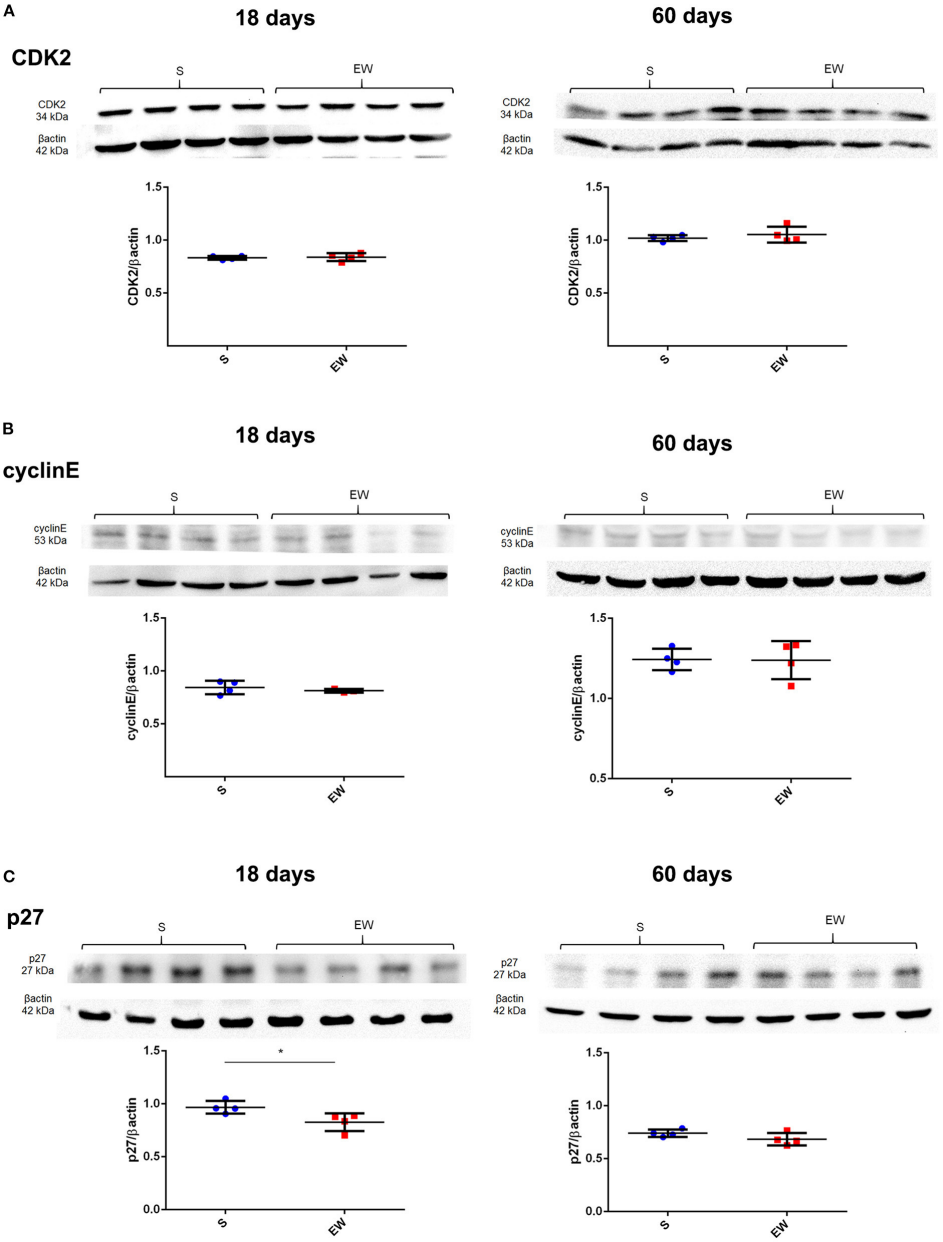
Early weaning effects on cell cycle regulators in the gastric mucosa. **(A–C)** Representative immunoblots for CDK2 **(A)**, cyclin E **(B)**, and p27 **(C)** and respective β-actin loads (four samples/group). Densitometry was used to compare the integrated optical density (IOD) of each isoform in each sample to β-actin control. Results are represented individually for S (blue) and EW (red) and by means ± SD ^*^p < 0.05 after Student's *t*-test.

As for cell cycle control, we verified that the EW did not alter the expression of *Cdk2, Ccne1, Cdkn1a*, and *Cdkn1b* at 18 and 60 days (Supplementary Figure 3A) and the encoded proteins CDK2 and cyclin E (Figures 3A,B). However, p27 levels decreased in EW animals at 18 days (*p* < 0.05), whereas no variation was detected at 60 days (Figure 3C).

The results above indicated, for the first time, that, during growth and aging, suckling and early weaning differentially affected the PI in active niches and the distribution of proliferative cells in the gland segments in a way that EW set and stabilized cell proliferation to the adult pattern immediately after feeding change, without the developmental variations described in S rats. Additionally, when niches were closely observed, a resilient proliferative activity remained higher at the base after EW, compared with the S group at 60 days. Such an area is a secondary compartment for epithelial proliferation (Han et al., 2019; Burclaff et al., 2020), and the residing zymogenic cell population is increased by EW (Teles Silva et al., 2020). Due to these combined data, we investigated some of the parameters that could be involved in cell trans-differentiation. Inflammation was not identified neither in morphological analyses (Supplementary Figure 2A) nor after gene profiling (*Nfkb1, Il1b*, and *Mmp9*, Supplementary Table 2) (Supplementary Figure 2B), except for *Nf*κ*b1* that increased in EW rats at 60 days (*p* < 0.05) (Supplementary Figure 2B). We also searched for genes related to metaplasia progression (*Mal2, Wfdc2, Mcm3*, and *Tacc2*) (Nozaki et al., 2008; Weis et al., 2014), and we noted that *Mal2* mRNA was significantly increased after EW (60 days) (*p* < 0.05) (Supplementary Figure 3B), whereas *Wfdc2* was reduced (18 days) (*p* < 0.001) (Supplementary Figure 3B). Because *Nf*κ*b1* and *Wfdc2* are related to cellular responses that indicate changes in tissue behavior, we also compared the intermediary samples collected at 30 days (Supplementary Figure 5) and studied the interaction of the feeding pattern and the age. We found that, for these genes, the effect of early weaning depends on the age (*p* < 0.05 for interaction after two-way ANOVA), as *Nf*κ*b1* increases progressively and *Wfdc2* varies according to the growth period (Supplementary Figure 5).

### TGF Beta Regulation

**Figure 4:**
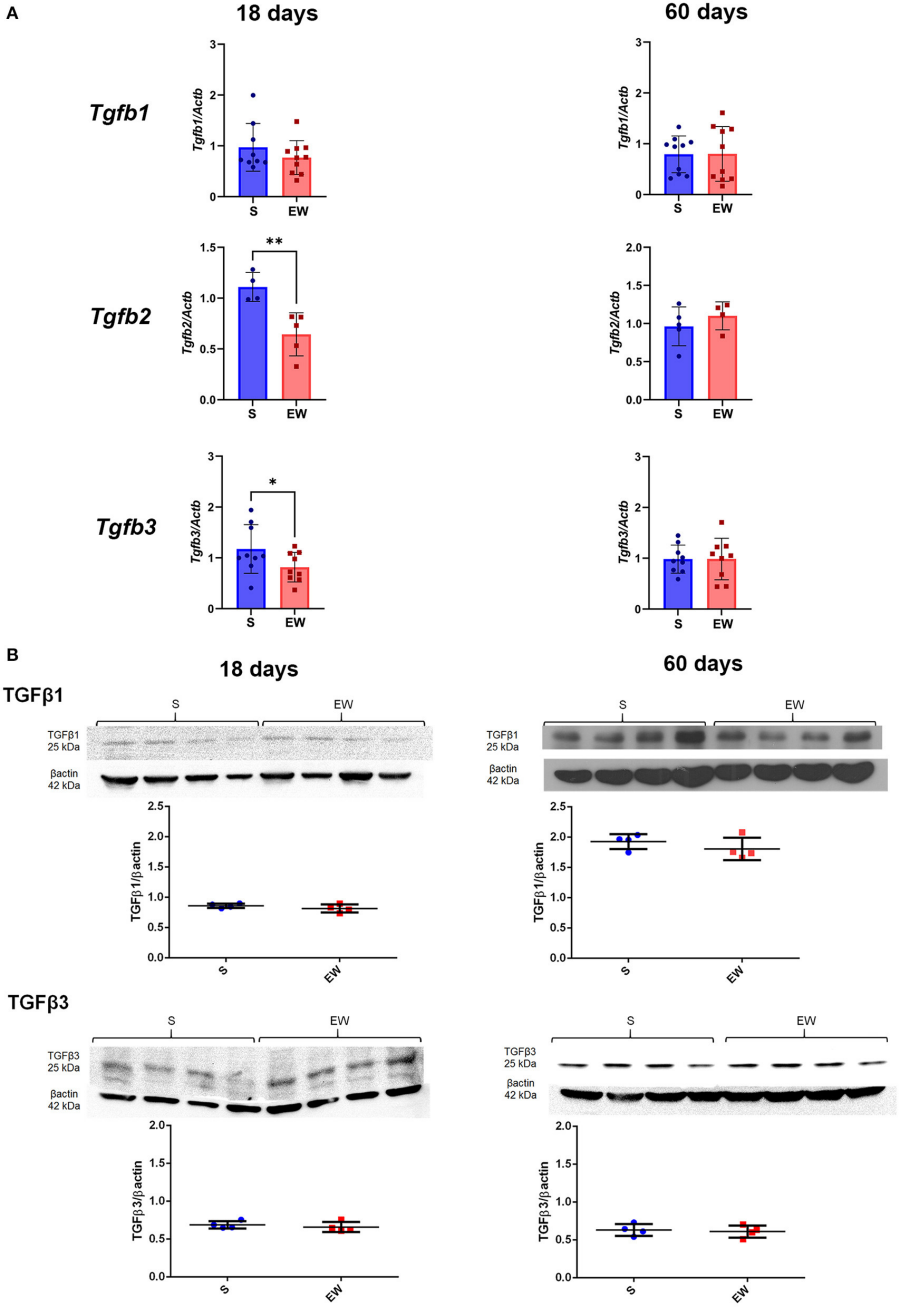
Differential effects of early weaning on gene expression and protein levels of the TGF beta isoforms. **(A)** *Tgfb1, Tgfb2*, and *Tgfb3* expressions in S (blue) and EW (red) groups at 18 and 60 days after RT-qPCR. **(B)** Representative immunoblots for TGFβ1 and TGFβ3 and respective β-actin loads (four samples/group). Densitometry was used to compare the integrated optical density (IOD) of each isoform in each sample to β-actin control. Results are represented individually for S (blue) and EW (red) and by means ± SD ^*^p < 0.05 and ^**^p < 0.01 after Student's *t*-test.

We observed that EW decreased the expression of *Tgfb2* (*p* < 0.01) and *Tgfb3* (*p* < 0.05) at 18 days, while *Tgfb1* was not altered (Figure 4A), whereas, at 60 days, these genes were not changed (Figure 4A). Because TGFβ1 and TGFβ3 are involved in cell proliferation control in the gastric mucosa (de Andrade Sá et al., 2008), we evaluated the peptide levels, and we noticed that both of them remained constant after EW at both ages (Figure 4B).

**Figure 5:**
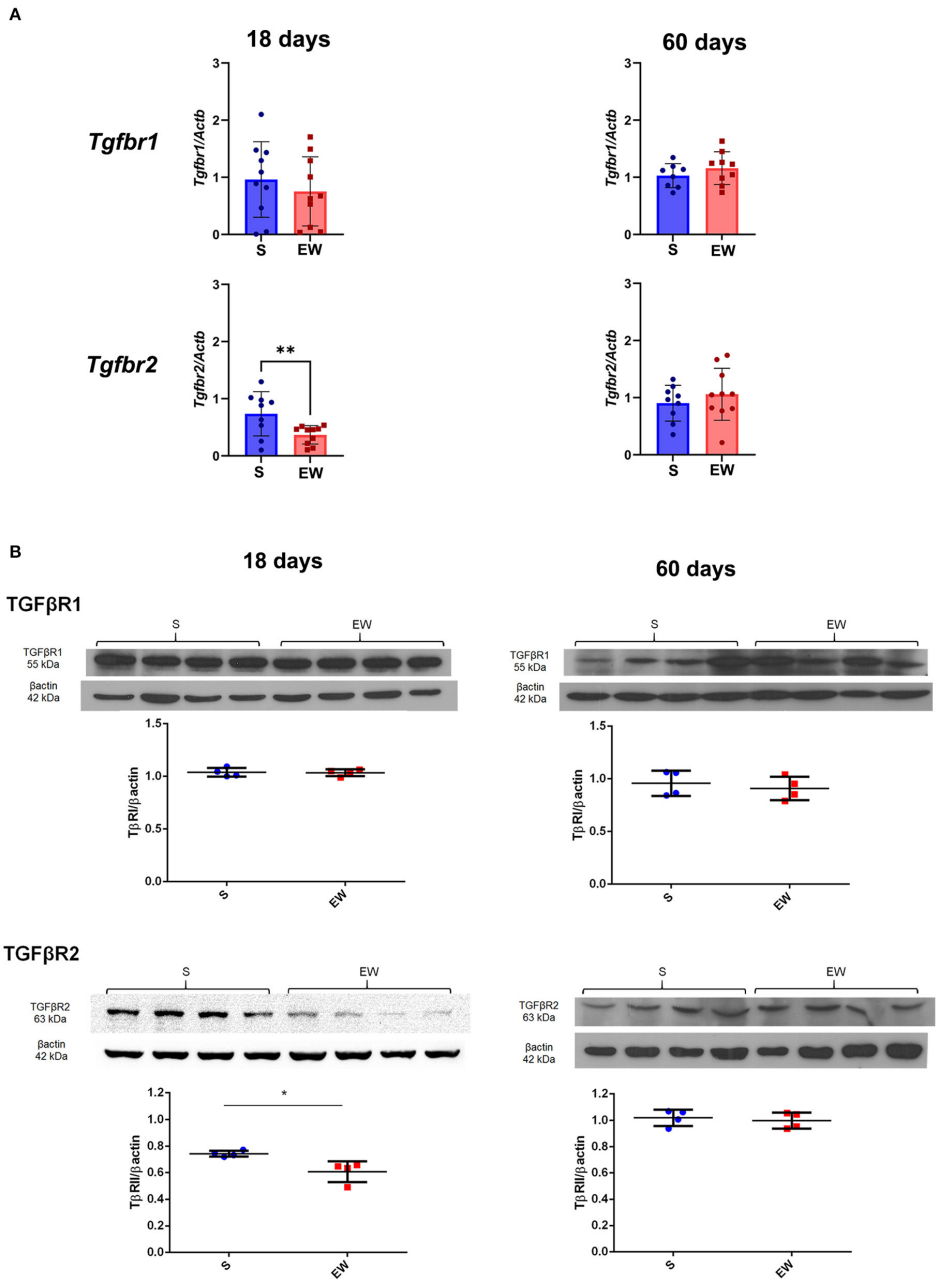
Differential effects of early weaning on gene expression and protein levels of the TGF beta receptors. **(A)** *Tgfbr1* and *Tgfbr2* expressions in S (blue) and EW (red) groups at 18 and 60 days after RT-qPCR. **(B)** Representative immunoblots for TGFβR1 and TGFβR2 and respective β-actin loads (four samples/group). Densitometry was used to compare the integrated optical density (IOD) of each receptor in each sample to β-actin control. Results are represented individually for S (blue) and EW (red) and by means ± SD ^*^p < 0.05 and ^**^p < 0.01 after Student's *t*-test.

We also found that EW did not modify the expression of *Tgfbr1*, but it reduced the expression of *Tgfbr2* (*p* < 0.01) (Figure 5A) and TβRII protein levels at 18 days (*p* < 0.05) (Figure 5B). At 60 days, genes and receptor proteins were unaltered (Figures 5A,B). As *Tgfbr2* is an important gene for the TGFβ pathway, besides checking the two hallmarks of the developmental period (18 days) and adulthood (60 days), we also compared the intermediary samples at 30 days (Supplementary Figure 5). We observed that expression is decreased by EW at this age (*p* < 0.05), and, in fact, the response of *Tgfbr2* to EW is age dependent (*p* < 0.05 for interaction after two-way ANOVA) (Supplementary Figure 5).

**Figure 6:**
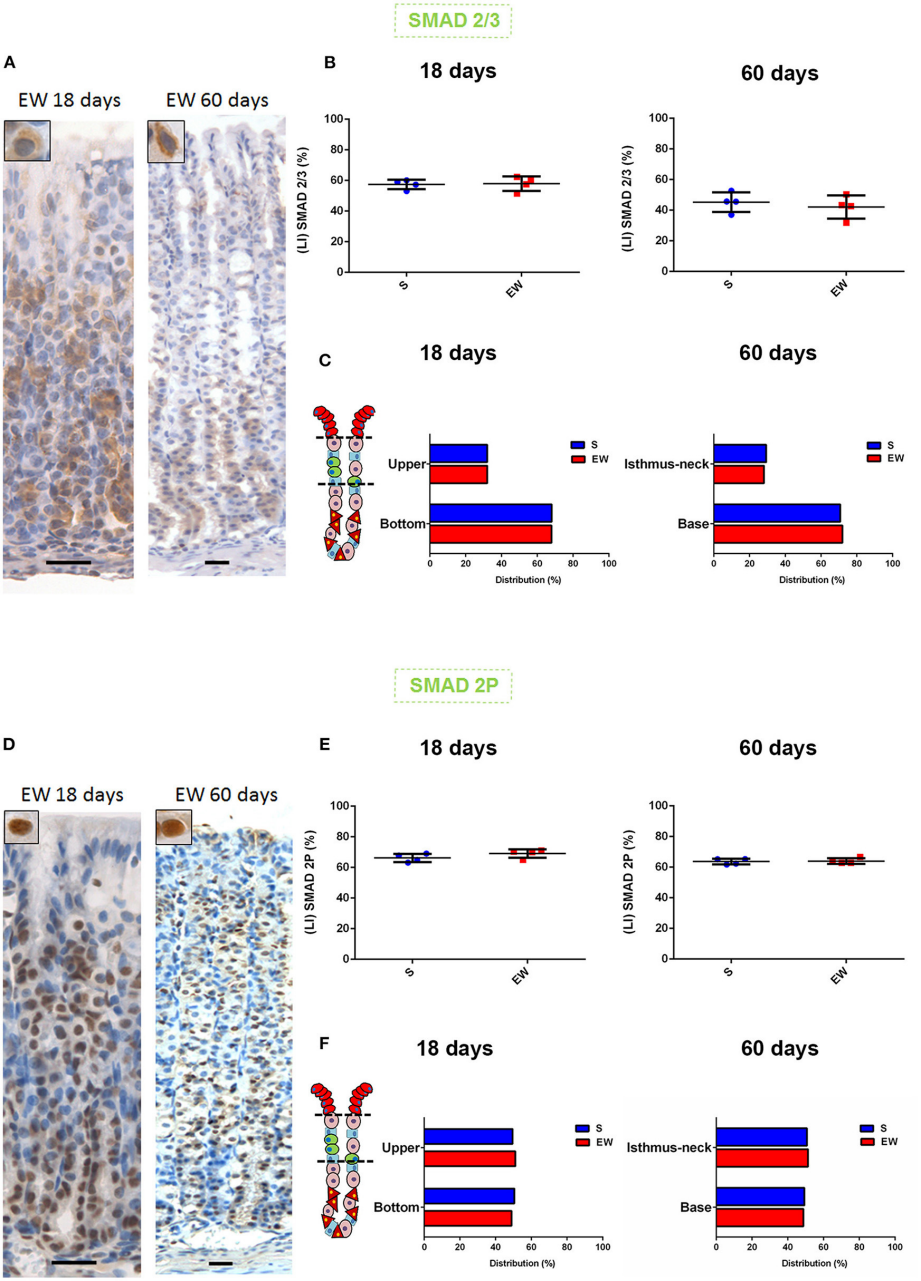
Early weaning and the distribution of Smads in the gastric mucosa **(A)**. **(D)** Representative photomicrographs: gastric mucosa at 18 and 60 days after immunolabeling for Smad 2/3 and Smad 2P, respectively. Scale bar: 25 μm. **(B,E)** Labeling index (LI) (%) for Smad 2/3 and Smad 2P, respectively, in S and EW rats (18 and 60 days). Results are represented individually and by means ± SD for S (blue) and EW (red). **(C,F)** Distribution of cells immunolabeled (%) for Smad 2/3 and Smad 2P, respectively, in the regions of the gastric gland in S and EW rats at 18 (as upper and bottom) and 60 (as isthmus neck and base) days. Bars represent the means for S (blue) and EW (red). Results were compared by Student's *t*-test according to the age studied.

TGFβ signals *via* Smad 2/3, which is phosphorylated (Smad 2P) and translocated to the nucleus to regulate the transcription (Massagué, 1998). We did not identify EW-induced changes in the labeling indices (LI) forSmad 2/3 (Figures 6A,B) (Supplementary Figure 4A) and Smad 2P (Figures 6D,E) (Supplementary Figure 4B), but, when we compared 18 and 60 days, we found a higher nuclear Smad 2P LI in pups than in adults (18 days: S = 87.7%; EW = 86.8%) (60 days: S = 76.2%; EW = 75.6%) (Supplementary Figure 4C). We also investigated the distribution of Smads in the mucosa, and in both ages and S and EW groups, we found the cytoplasmic staining for Smad 2/3 along the gland (Figure 6A), with a higher LI at the base (bottom) (Figure 6C). In contrast, the Smad 2P nuclei (Figure 6D) were equally distributed in the isthmus neck (upper) and the base (bottom) (Figure 6F). Our results did not suggest changes in Smads distribution; however, the presence of Smads 2/3 and Smad 2P along the whole gland indicates the continuous activity of TGFβ on the different cell types, which might contribute directly to cell cycle control.

## Discussion

Early weaning activates stress responses in rat pups, which are induced both by the interruption of milk-born molecules ingestion and maternal separation (Ghizoni et al., 2014; Zulian et al., 2017). During early weaning, the rapid regulation of corticosterone and EGFR cascades coordinate the acceleration of gastric maturation (Osaki et al., 2010, 2011; Osaki and Gama, 2013; Ghizoni et al., 2014; Zulian et al., 2017; Teles Silva et al., 2020). Because the triggers for cell cycle control and differentiation have been previously evaluated in pups, in the current study, we investigated the dynamics of early weaning effects on the stomach in terms of cell proliferation and expression of molecular markers that could characterize the latency of changes and identify any scenario for induction of injuries during growth and aging.

In the developing gastric mucosa, cell proliferation targets growth, whereas in adults, the process is important for gland maintenance. We observed that, depending on the dietary pattern practiced during postnatal development, the gastric proliferative activity was differentially regulated, and, in that sense, the rats that had been breastfed regularly showed a peak of Ki-67 labeling at 30 days, whereas the EW group increased the PI at 18 days, keeping it high until 60 days. Such difference between S and EW rats might be dependent on the balance of signaling cascades that would trigger an immediate response to EW that would be followed by a modulation, making the gastric mucosa less responsive to the developmental effects that were detected in S animals.

The different dynamics of gastric cell proliferation between S and EW was also reflected in the distribution of proliferative cells in gland segments, and, early at 18 days, in EW mucosa, Ki-67-labeled cells concentrated at upper and middle areas, which turn to be the isthmus and part of the neck in the mature mucosa. These results agreed with reports that demonstrated that early weaning anticipates the organization of gastric gland (Gama and Alvares, 2000; Osaki et al., 2010; Ghizoni et al., 2014). The mechanisms involved in cellular positioning in the mucosa have not been described yet; however, corticosterone participates in this process. When RU486 (glucocorticoid receptor antagonist) is administered to early-weaned developing animals, the PI is kept at a high level, but Ki-67 immuno-stained cells turn back to the distribution observed in controls (Ghizoni et al., 2014), which means that they get spread along the gland. Also, after weaning and throughout adulthood, there is an establishment of the glandular organization, in which the isthmus houses the highest proliferation rates in comparison to other gland areas. Interestingly, until 2019, there was consensus that the isthmus-neck region was responsible for maintaining the entire epithelial population in the mucosa (Karam, 1993; Burclaff and Mills, 2018). However, recent studies (Han et al., 2019; Burclaff et al., 2020) have demonstrated the compartmentalization, with two independent stem cell populations at the isthmus and the base. It is not yet clear how this segregation occurs during postnatal development and whether the feeding pattern could influence the process. Our results suggest that, in the third postnatal week, under regular breastfeeding, Ki-67 immunolabeled cells are found along the epithelium, indicating the equal participation of the gland, without a clear distinction of niches. However, when the feeding pattern changes with EW, cells are pushed to the upper and middle segments, characterizing the active and slow cycling niches. Previously, we demonstrated that EW immediately expands the population of mucous neck and zymogenic cells (Teles Silva et al., 2020), possibly through the increased cell proliferation in the main niche. But, at 60 days, when cell proliferation targets mucosa maintenance and the proliferative indices are constant in the EW group, the doubling rate at the base niche would prime the gland to expand the zymogenic population, as described before (Teles Silva et al., 2020). Because such response might be dependent on molecular mechanisms that were not apparent through morphology, next, we evaluated the molecules involved in the TGF beta pathway and the cell cycle.

Our results indicated that the TGF beta cascade was differentially altered by EW when gene expression and protein levels were evaluated in a way that *Tgfb2, Tgfb3*, and *Tgfbr2* mRNA were reduced at 18 days, and, from these genes, only receptor TβRII was equally decreased. Also, we did not detect evidence of changes in Smads signaling, and such observation could be related to the timing of sample collection after EW since Smads move into the nucleus rapidly (de Andrade Sá et al., 2008). However, the reduction of *Tgfbr2* and TβRII suggested their function on gastric growth, especially at 18 days, characterizing an immediate response. The role of TβRII in cell proliferation has been reported *in vivo* and *in vitro* (Okamoto et al., 1994; Chang et al., 1997; Heldin et al., 1997; Akhurst and Balmain, 1999; Beck et al., 2003; de Andrade Sá et al., 2008), and its decrease can be related to the high proliferation at 18 days, as discussed before (Osaki et al., 2010; Ghizoni et al., 2014; Zulian et al., 2017). Conversely, the similarity between S and EW groups at 60 days was parallel with the PI at this age, and, so, the stability of TGF beta cascade in adults would be part of cell proliferation adaptive mechanisms triggered after EW.

Among cell cycle regulatory molecules, none of the genes were affected, but p27 protein levels behaved similarly to the TGF beta family with a decrease after EW only at 18 days. Different studies demonstrated that, in the gastric epithelia p27 expression, synthesis and degradation are sensitive to changes in the feeding pattern (de Andrade Sá et al., 2008; Ogias et al., 2010; Osaki and Gama, 2013; Fiore et al., 2014). Low p27 concentration is a prevalent condition for tumor growth, and its deficiency correlates with gastric (Kuzushita et al., 2005) and endometrial cancers (Lecanda et al., 2009; Pavlides et al., 2016). Conversely, high p27 leads to tumor suppression in gastric cells (Liu et al., 2019). We did not find evidence of lesions after EW, but the low levels at 18 days corroborated previous data that showed that p27 can be regulated by different feeding patterns, and it controls cell proliferation in the gastric epithelium (de Andrade Sá et al., 2008; Ogias et al., 2010; Osaki and Gama, 2013; Fiore et al., 2014).

Currently, it is known that the spasmolytic polypeptide expressing metaplasia (SPEM) represents an important step after gastric inflammation, and it involves the trans-differentiation of zymogenic cells into SPEM (Quante et al., 2010; Goldenring et al., 2011; Mills and Sansom, 2015; Radyk et al., 2018; Burclaff et al., 2020). Although some molecules identify metaplasia (Nozaki et al., 2014), both their expression and the inflammatory markers have not been associated with development or feeding condition. By combining the morphological analyses, the panel of genes herein studied, and previous results that indicated the maintenance of the GSII^+^GIF^+^ transition compartment after EW (Teles Silva et al., 2020), we can infer that soon after early weaning (18 days), different factors, which include EGFR pathways, TGF beta and corticosterone activity would downregulate the transcription of genes that enable inflammation and cell disorders. In that way, the gastric mucosa architecture would be protected to allow growth. As the proliferative rate was kept constant and only one parameter (Mal 2 expression) was augmented at 60 days, further studies are necessary to demonstrate whether EW primes the gastric mucosa for lesions and metaplasia.

In summary, we demonstrated that, in rat pups, early weaning increased gastric cell proliferation and growth that were not concomitant with inflammation and metaplasia, and such responses involved the rearrangement of proliferative cells in the gland, and the downregulation of TβRII and p27. Moreover, as we traced the later effects at 30 and 60 days, we found that the constant proliferative indices after EW did not follow the variation observed in S rats, suggesting that the changes induced by breastfeeding interruption altered the proliferative responses in the gastric epithelium. Such effect might be derived from the early modulation of TGF beta signaling on cell cycle control.

## Data Availability Statement

The raw data supporting the conclusions of this article will be made available by the authors, without undue reservation.

## Ethics Statement

The animal study was reviewed and approved by Ethical Committee on Animal Use (CEUA ICB) at Institute of Biomedical Sciences, University of São Paulo.

## Author Contributions

KM, IR, and GP collected and processed the samples and analyzed the data. KM, IR, GP, and PG planned the experiments and wrote and reviewed the manuscript. KM and IR prepared the figures. PG planned the study, applied for financial support, and reviewed the data and figures. All authors contributed to the article and approved the submitted version.

## Funding

This study was supported by São Paulo Research Foundation (FAPESP) (Grants 2014/21449-9; 2018/07782-8; fellowship 2018/05064-0; 2021/09522-6) by Coordenação de Aperfeiçoamento de Pessoal de Nível Superior (CAPES code 001) and by Conselho Nacional de Desenvolvimento Científico e Tecnológico (CNPq, Brazil, 134005/2015-5).

## Conflict of Interest

The authors declare that the research was conducted in the absence of any commercial or financial relationships that could be construed as a potential conflict of interest.

## Publisher's Note

All claims expressed in this article are solely those of the authors and do not necessarily represent those of their affiliated organizations, or those of the publisher, the editors and the reviewers. Any product that may be evaluated in this article, or claim that may be made by its manufacturer, is not guaranteed or endorsed by the publisher.
