# Supplementary material for: Lifelong Adaptation of Gastric Cell Proliferation and Mucosa Structure to Early Weaning-Induced Effects
Source: Front Physiol. 2021 Sep 13;12:721242. doi: 10.3389/fphys.2021.721242 (PMC8475651; doi:10.3389/fphys.2021.721242)
Supplement: Supplementary file 1 [file Data_Sheet_1.docx]

***Supplementary Material***

**Lifelong adaptation of gastric cell proliferation and mucosa structure to early weaning-induced effects**

Kethleen Mesquita da Silva^#^, Isadora Campos Rattes^#^, Gizela Maria Agostini Pereira, Patrícia Gama*

Department of Cell and Developmental Biology, Institute of Biomedical Sciences, University of Sao Paulo, 05508-000, Sao Paulo, SP, Brazil.

# authors share first- authorship

*Correspondence: patgama@usp.br; +55 11 30917303

*Supplemental material*

*Tables S1 and S2*

*Figures S1- S5*

**Suppl. Table 1. Antibodies used for immunoblot and immunohistochemistry.**

| Primary antibody | Concentration  (μg/mL) | Secondary antibody*^a^* | Concentration  (μg/mL) |
| --- | --- | --- | --- |
| Beta-actin mouse monoclonal  (Sigma) | 1:10000 | Goat anti-mouse | 0.53 |
| TGFβ1 rabbit polyclonal  (Santa Cruz) | 1 | Goat anti-rabbit | 0.53 |
| Ki-67 rabbit monoclonal  IHC reaction (Abcam) | 1:50 | Goat anti-rabbit | 11 |
| TGFβ3 rabbit polyclonal  (Santa Cruz) | 1 | Goat anti-rabbit | 0.53 |
| TβR1 rabbit polyclonal  (Santa Cruz) | 1 | Goat anti-rabbit | 0.53 |
| TβR2 rabbit polyclonal  (Santa Cruz) | 1 | Goat anti-rabbit | 0.53 |
| SMAD 2/3 rabbit polyclonal  (Millipore) | 1:750 | Goat anti-rabbit | 0.8 |
| SMAD 2/3 rabbit polyclonal  IHC reaction (Millipore) | 1:100 | Goat anti-rabbit | 32 |
| SMAD 2P rabbit polyclonal  (Millipore) | 1:750 | Goat anti-rabbit | 0.8 |
| SMAD 2P rabbit polyclonal  IHC reaction (Millipore) | 1:100 | Goat anti-rabbit | 32 |
| CDK2 rabbit polyclonal  (Santa Cruz) | 1 | Goat anti-rabbit | 0.53 |
| cyclinE rabbit polyclonal  (Santa Cruz) | 1 | Goat anti-rabbit | 0.53 |
| p27 rabbit polyclonal  (Santa Cruz) | 1 | Goat anti-rabbit | 0.53 |

*^a^* Conjugated with peroxidase (Jackson Labs).

**Suppl. Table 2 – TaqMan® probe assays were used for RT-qPCR, according to the encoded protein and the gene group.**

| Group | Gene | Encoded protein | TaqMan® assay |
| --- | --- | --- | --- |
| Endogenous control | ***Actb*** | Beta-actin | Rn00667869_m1 |
| Growth factor | ***Tgfb1*** | TGFβ1 | Rn00572010_m1 |
| Growth factor | ***Tgfb2*** | TGFβ2 | Rn00579674_m1 |
| Growth factor | ***Tgfb3*** | TGFβ3 | Rn00565937_m1 |
| TGFβ receptor | ***Tgfbr1*** | TGFβR1 | Rn00562811_m1 |
| TGFβ receptor | ***Tgfbr2*** | TGFβR2 | Rn00579682_m1 |
| Cell cycle | ***Cdk2*** | CDK2 | Rn01529540_m1 |
| Cell cycle | ***Ccne1*** | cyclinE | Rn01457762_m1 |
| Cell cycle | ***Cdkn1a*** | p21 | Rn00589996_m1 |
| Cell cycle | ***Cdkn1b*** | p27 | Rn00582195_m1 |
| Inflammation | ***Nfkb1*** | NFκb1 | Rn01399583_m1 |
| Inflammation | ***Il1b*** | IL1B | Rn00580432_m1 |
| Inflammation | ***Mmp9*** | MMP9 | Rn00579162_m1 |
| Metaplasic cell | ***Mal2*** | MAL 2 | Rn01503894_m1 |
| Metaplasic cell | ***Wfdc2*** | WFDC2 | Rn00687489_m1 |
| Metaplasic cell | ***Tacc2*** | TACC2 | Rn01506428_m1 |
| Metaplasic cell | ***Mcm3*** | MCM3 | Rn01511840_m1 |

Supplemental Figures

**
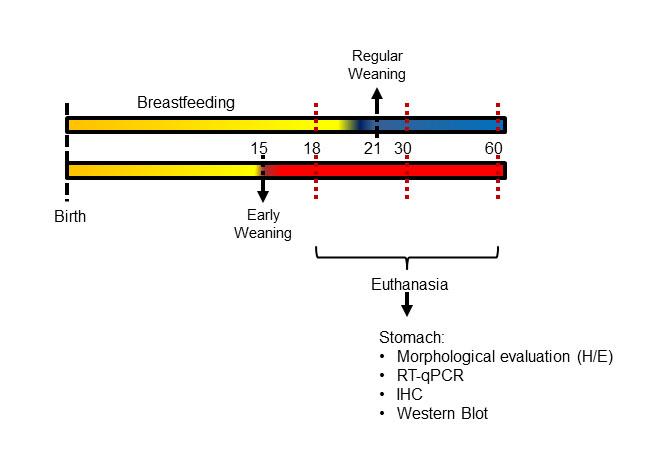
**

Figure S1. Experimental design. At 15 days rats were divided into control (regular suckling- weaning periods) (S) and early-weanling (EW) groups. Under anesthesia, the stomach was collected at 18, 30 and 60 d to be fixed in 10% formaldehyde for morphological evaluation and immunohistochemistry, which were studied under light microscopy, and the gastric mucosa was also scraped for RNA or protein isolation for RT qPCR and western blot analysis, respectively.

**
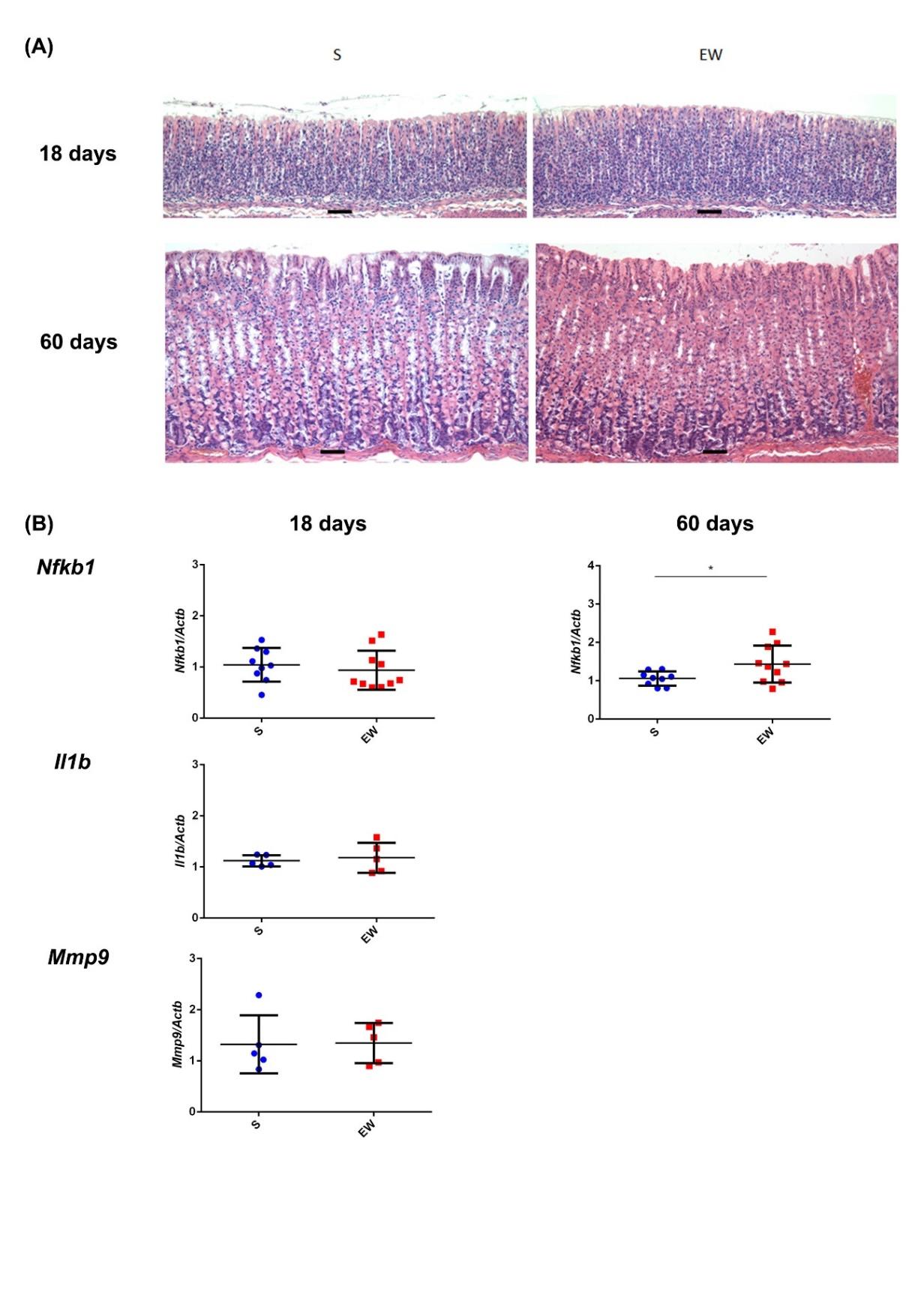
**

**Figure S2.** Early weaning did not induce inflammation in the gastric mucosa, but it changed gene expression in adult rats. (*A*) Representative photomicrographs of the gastric mucosa in S and EW (18 and 60 d). Sections were stained with HE. Scale bar: 25 μm. (*B*) From the seven genes studied (Table S2), only *Nfkb1, Il1b* and *Mmp9* were detected in S and EW groups at 18 days. At 60 d, only *Nfkb1* mRNA amplified after RT-qPCR. Results are represented individually and by means ± SD for S (blue) and EW (red). Samples were compared after Student's *t* test. * *P* <0.05.

**
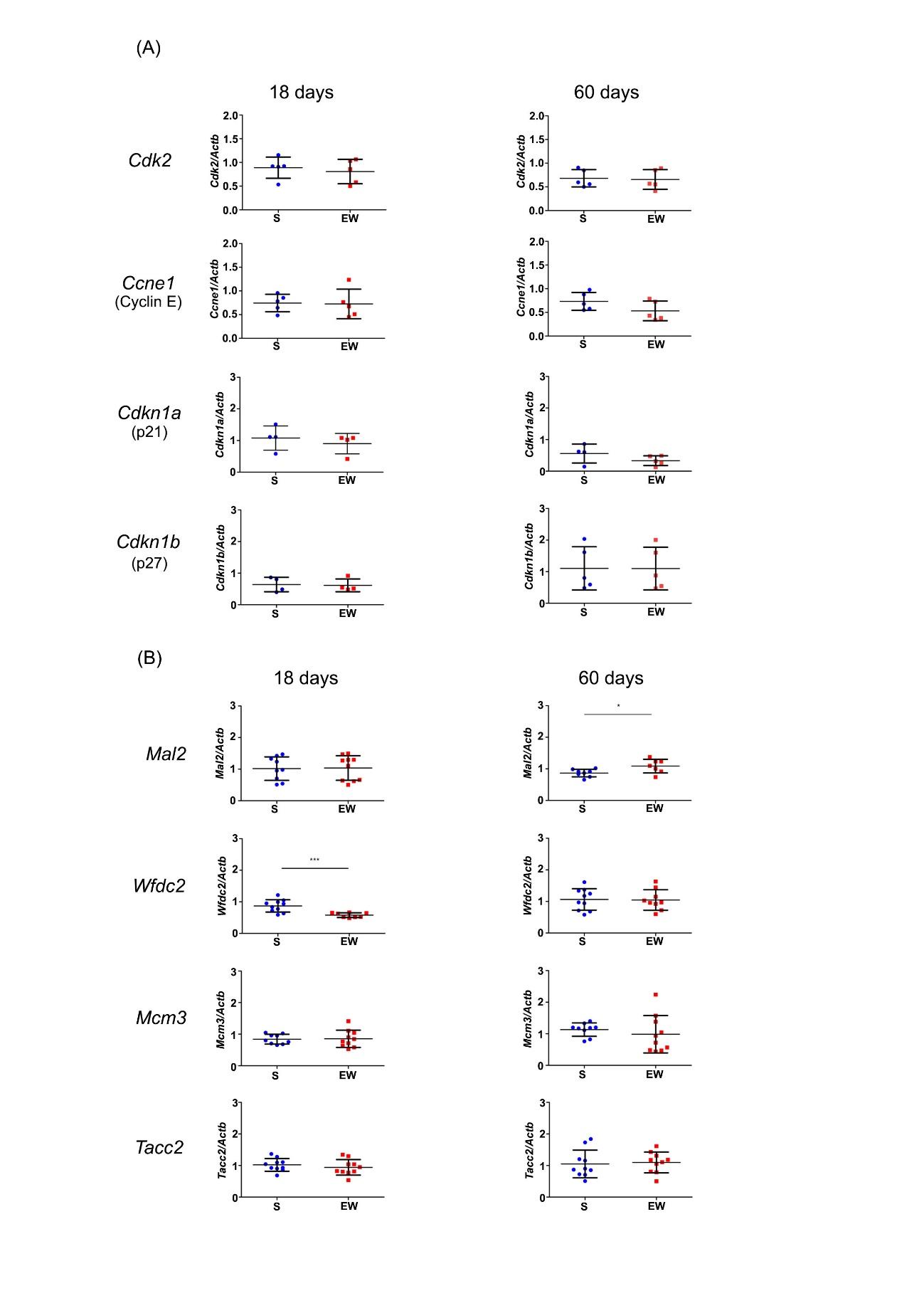
**

**Figure S3.** Effects of early weaning on gene expression of cell cycle regulators and metaplasia in the gastric mucosa. (*A*), (*B*) Expression of *Cdk2, Ccne1, Cdkn1a, and Cdkn1b* (*A*) and *Mal2, Wfdc2, Mcm3, Tacc2* (*B*) in S and EW groups (18 and 60 d) after RT‐qPCR. Results are represented individually and by means ± SD for S (blue) and EW (red). Samples were compared after Student's *t* test. * *P* <0.05.

**
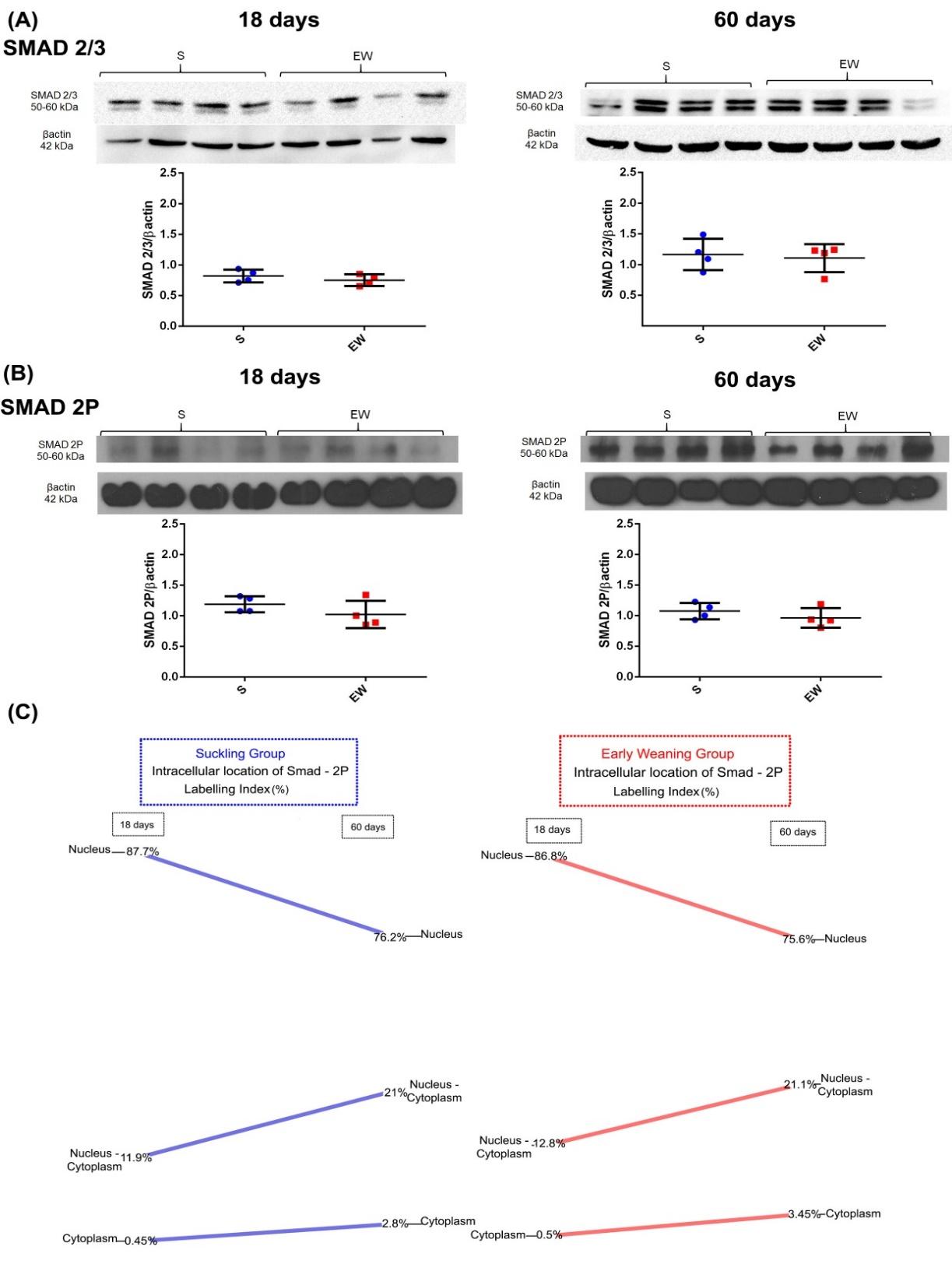
**

**Figure S4.** Early weaning did not affect the Smad protein levels. (*A*) (*B*) Representative immunoblots for Smad 2/3 (A) and Smad 2P (*B*) and respective β-actin loads (four samples/group). Densitometry was used to compare the integrate optical density (IOD) of each isoform in each sample to β-actin control. Results are represented individually for S (blue) and EW (red) and by means ± SD. Protein levels were compared by Student's t test according to the age. (*C*) Slope graph for the intracellular location of Smad 2P in S (blue) and EW (red) groups from 18 to 60 d. The analysis showed that at 60 days the nuclear Smad 2P distribution was lower than at 18 d.


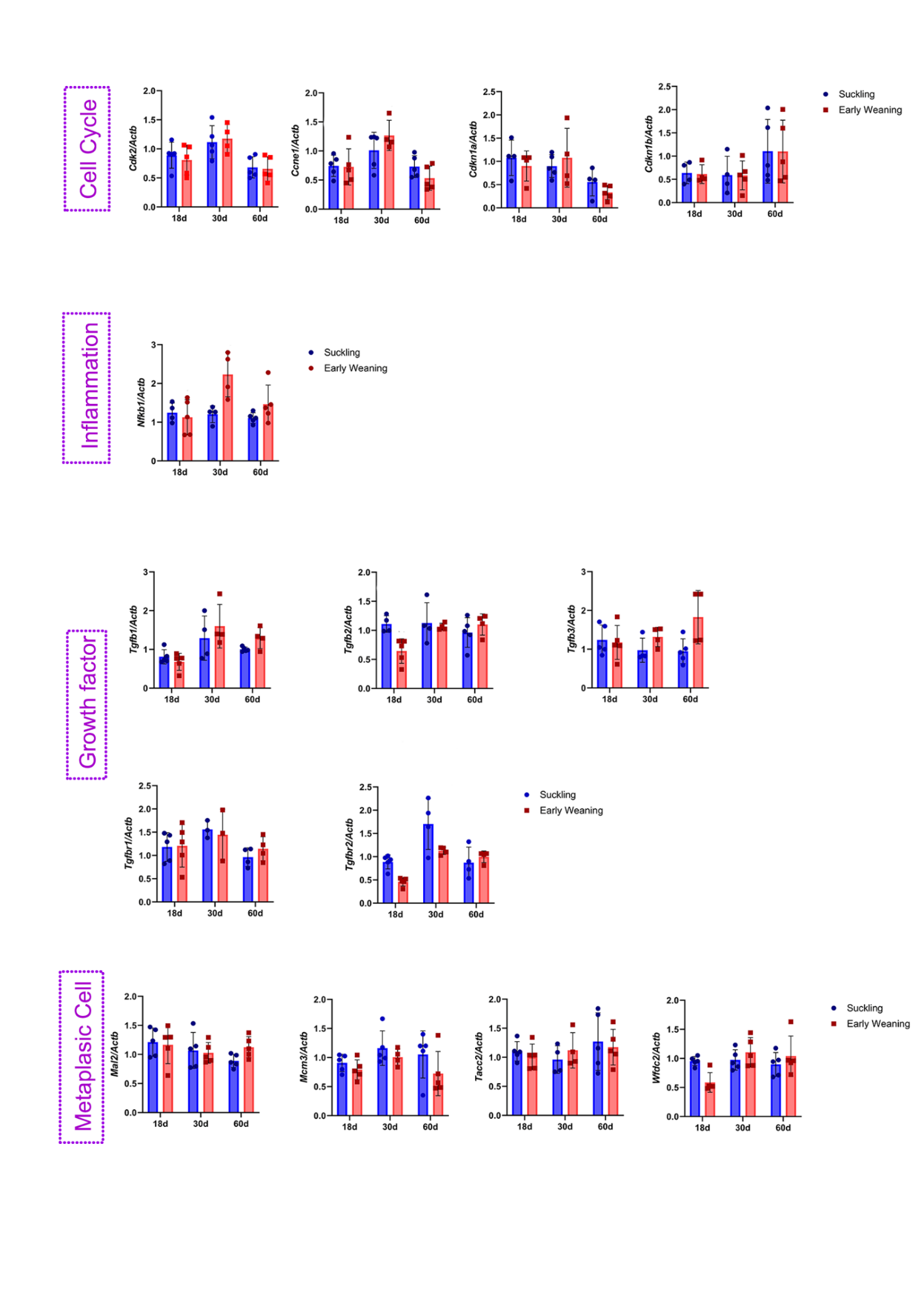


**Figure S5.** Effects of early weaning on gene expression of cell cycle regulators, inflammation, growth factors and metaplasia in the gastric mucosa of 30-d-old rats. Expression of *Cdk2, Ccne1, Cdkn1a, and Cdkn1b, NfκB, Tgfb1, Tgfb2, Tfgbr1, Tgfbr2* and *Mal2, Wfdc2, Mcm3, Tacc2* in S and EW groups of 30 days after RT‐qPCR. Results are represented individually and by means ± SD for S (blue) and EW (red). Samples were compared after Two-way ANOVA, and interaction between feeding pattern (suckling and early weaning) and age was significant (*P <* 0.05) for *NfκB*, *Wfdc2* and *Tgfbr2.*
